# Supplementary material for: Dynamic transcriptomic profiles of zebrafish gills in response to zinc supplementation
Source: BMC Genomics. 2010 Oct 11;11:553. doi: 10.1186/1471-2164-11-553 (PMC3091702; doi:10.1186/1471-2164-11-553)
Supplement: Additional file 2 — Interactive Direct Interaction Network representing the molecular interactions between zinc, copper, iron, calcium and proteins encoded by transcripts changed by zinc supplementation. Mini web-site containing index.html and hyperlinked pages in subdirectory describing a Direct Interaction Network automatically generated based on curated interactions contained within the proprietary PathwayArchitect database. Ovals represent proteins and the circles symbolize metal ions. Objects are coloured by their abundance in zebrafish at the time-point they were significantly different from the control is a scale from -4 fold (dark green) to +4 fold (dark red). Where significant differences were found at more than one time-point, the colour overlay shows expression at the first instance. Dark blue squares denote 'binding', and light blue squares 'expression'; green squares stand for 'regulation', green diamonds for 'metabolism', and green circles for 'promoter binding'. Arrow heads indicate directionality of the interaction where annotated. All nodes and edges can be further interrogated by selecting the relative area of the image. [file 1471-2164-11-553-S2.zip › PathwayArchitect Zn xs DIN/680310.html]

# REGULATION:

|  |  |
| --- | --- |
| Type | REGULATION |
| Effect | Positive |


---

|  |  |
| --- | --- |
| Score | 0 |


---

|  |  |
| --- | --- |
| Reference Count | 5 |


---

|  |  |
| --- | --- |
| Mechanism | Unknown |


---

|  |  |
| --- | --- |
| Reference:0 || Sentence | "We have examined the effects of the iron(III) chelator deferroxamine (DFO) and iron (FeCl(3)) on UVB (290-320 nm)-induced activator protein 1 (AP-1) signaling." |
| PMID | 10545417 |
| Year | 1999 |
| Species | Human |
| Journal | Carcinogenesis |
| RefScore | 0 |
| Source | PArchNLP |
  |
|  |  |


---

|  |  |
| --- | --- |
 Reference:1 || Sentence | "These results indicate that UVB-induced AP-1 activation may be in part due to oxidant effects of UVB and intercellular iron." |
| PMID | 10545417 |
| Year | 1999 |
| Species | Human |
| Journal | Carcinogenesis |
| RefScore | 1 |
| Source | PArchNLP |
  ||  |  |


---

|  |  |
| --- | --- |
 Reference:2 || Sentence | "Deferoxamine (DFO), an iron chelator, synergistically enhanced the PA coal-induced AP-1 activity, but inhibited NFAT activity." |
| PMID | 12397016 |
| Year | 2002 |
| Species | Mouse |
| Journal | Am J Respir Cell Mol Biol |
| RefScore | 2 |
| Source | PArchNLP |
  ||  |  |


---

|  |  |
| --- | --- |
 Reference:3 || Sentence | "We have found that iron transactivated both AP-1 and NFAT, and DFO further enhanced iron-induced AP-1 activation but inhibited NFAT." |
| PMID | 12397016 |
| Year | 2002 |
| Species | Mouse |
| Journal | Am J Respir Cell Mol Biol |
| RefScore | 1 |
| Source | PArchNLP |
  ||  |  |


---

|  |  |
| --- | --- |
 Reference:4 || Sentence | "We have shown by the electrophoretic mobility shift assay that paraquat, together with low concentrations of chelated iron (Fe++/DETAPAC), induced the activation of transcription factor AP-1 binding activity to DNA." |
| PMID | 10195331 |
| Year | 1999 |
| Species | Rat |
| Journal | J Neural Transm |
| RefScore | 1 |
| Source | PArchNLP |
  |


---

|  |  |
| --- | --- |
